# Lactate versus non-lactate metabolic acidosis: a retrospective outcome evaluation of critically ill patients

**DOI:** 10.1186/cc3987

**Published:** 2006-02-10

**Authors:** Kyle J Gunnerson, Melissa Saul, Shui He, John A Kellum

**Affiliations:** 1 Assistant Professor, VCURES (Virginia Commonwealth University Reanimation Engineering Shock Center) Laboratory, Departments of Anesthesiology/Critical Care and Emergency Medicine, Medical College of Virginia/Virginia Commonwealth University, 1200 East Broad Street, Richmond, VA, 23298, USA; 2 Director, Clinical Research Informatics Service, University of Pittsburgh, 450 Scaife Hall, 200 Lothrop St. Pittsburgh, PA, 15213, USA; 3 Research Assistant, Department of Biostatistics, University of Pittsburgh, Graduate School of Public Health, Crabtree Hall, Pittsburgh, PA, 15213, USA; 4 Professor, CRISMA (Clinical Research, Investigation, and Systems Modeling of Acute illness) Laboratory, Department of Critical Care Medicine, University of Pittsburgh, 608, Scaife Hall, 3550 Terrace Street, Pittsburgh, PA 15261, USA

## Abstract

**Introduction:**

Acid–base abnormalities are common in the intensive care unit (ICU). Differences in outcome exist between respiratory and metabolic acidosis in similar pH ranges. Some forms of metabolic acidosis (for example, lactate) seem to have worse outcomes than others (for example, chloride). The relative incidence of each type of disorder is unknown. We therefore designed this study to determine the nature and clinical significance of metabolic acidosis in critically ill patients.

**Methods:**

An observational, cohort study of critically ill patients was performed in a tertiary care hospital. Critically ill patients were selected on the clinical suspicion of the presence of lactic acidosis. The inpatient mortality of the entire group was 14%, with a length of stay in hospital of 12 days and a length of stay in the ICU of 5.8 days.

**Results:**

We reviewed records of 9,799 patients admitted to the ICUs at our institution between 1 January 2001 and 30 June 2002. We selected a cohort in which clinicians caring for patients ordered a measurement of arterial lactate level. We excluded patients in which any necessary variable required to characterize an acid–base disorder was absent. A total of 851 patients (9% of ICU admissions) met our criteria. Of these, 548 patients (64%) had a metabolic acidosis (standard base excess < -2 mEq/l) and these patients had a 45% mortality, compared with 25% for those with no metabolic acidosis (*p *< 0.001). We then subclassified metabolic acidosis cases on the basis of the predominant anion present (lactate, chloride, or all other anions). The mortality rate was highest for lactic acidosis (56%); for strong ion gap (SIG) acidosis it was 39% and for hyperchloremic acidosis 29% (*p *< 0.001). A stepwise logistic regression model identified serum lactate, SIG, phosphate, and age as independent predictors of mortality.

**Conclusion:**

In critically ill patients in which a measurement of lactate level was ordered, lactate and SIG were strong independent predictors of mortality when they were the major source of metabolic acidosis. Overall, patients with metabolic acidosis were nearly twice as likely to die as patients without metabolic acidosis.

## Introduction

An inconsistent relationship has been reported between acid–base abnormalities, their treatment, and outcomes [1-6] in critically ill patients. Some studies have suggested an independent association between low pH or standard base excess (SBE) and mortality [7-9], whereas others have not [1,10].

We have recently shown that hemodynamically stable rats with sepsis become hypotensive after infusion of dilute HCl [11]. Similarly we have found decreased survival time in rats with elevated serum chloride levels that were exposed to lethal doses of lipopolysaccharide [12], as well as different cytokine responses to acidosis induced by lactate or HCl in cell cultures [13]. We therefore propose that the etiologic anion of metabolic acidosis (lactate, chloride, or others) may be a more important determinant of outcome than the pH itself.

Although lactic acidosis has attracted considerable study in critically ill patients, metabolic acidosis can result from a variety of conditions [14]. Although the existing literature does not suggest a strong relationship between the type of acidosis and outcome, traditional methods of classifying and analyzing acid–base abnormalities have significant limitations, especially in critically ill patients [15]. In particular, studies have usually failed to identify the effects that causative anions (for example lactate, chloride, or all others) have on their resulting pH and SBE. Data may be reported as 'non-lactate metabolic acidosis' or 'anion gap metabolic acidosis' without identifying a predominant source. By using a physical–chemical approach [16-18] to quantify unmeasured anions accurately, coupled with multivariable logistic regression, we believe that a more rigorous characterization of acid–base disorders and their clinical significance can be achieved. Our objective was to determine the effect of different etiologies of metabolic acidosis on hospital mortality and length of stay in critically ill patients suspected of having lactic acidosis. The hypothesis was that the various etiologies of metabolic acidosis have unique mortality and morbidity rates associated with them.

## Materials and methods

### Rationale

Our intent was to identify a cohort of patients in which lactic acidosis was suspected but for whom other acid–base abnormalities might be present. Focusing on this group of patients permitted us to limit our analysis to a subgroup with a high incidence of metabolic acidosis and a high risk of death. Describing the incidence and impact of various forms of acidosis in this cohort is potentially more clinically relevant than in the entire intensive care unit (ICU) population.

We divided this cohort into two groups depending on the presence or absence of a metabolic acidosis, where SBE < -2 mEq/l determined metabolic acidosis. The metabolic acidosis group was further subdivided into groups depending on the anion contributing to the majority of the acidosis.

### Data abstraction

After approval from the Investigational Review Board of the University of Pittsburgh we searched the database of Medical Archival Systems Inc. (Pittsburgh, PA, USA) from 1 January 2001 to 30 June 2002 for all ICU admissions at the University of Pittsburgh Medical Center, Oakland, PA. We selected cases on the following criteria. First, clinicians caring for each patient suspected the presence of lactic acidosis and obtained an analysis for arterial blood gas (ABG) and serum lactate. In the hospitals included in this study, to obtain a lactate level a physician has to order one specifically. There are no routine, standing orders or protocols for lactate measurement. Thus, blood lactate measurements are usually obtained only when there is a suspicion of lactic acidosis. Next, to account for all ions, electrolytes had to be drawn within four hours (0.8 ± 2, mean ± SD) hours), and calcium, magnesium and phosphorus within 24 hours (mean 1.3 ± 4.6 hours) of the referenced ABG and an albumin level at some time during the hospitalization. If more than one albumin value was recorded, the lowest value was used to avoid spurious elevations due to possible resuscitation with albumin. If the patient had more than one ABG that fitted these criteria, the ABG with the highest lactate level was used. Finally, if there was more than one data set of electrolytes that fitted around the index ABG, the data set with an absolute time frame closest to the indexed ABG was used. Data could be collected at any time that the patient fitted these criteria. A few patients might have actually had the highest lactate level while in the Emergency Department or on the general wards; however, we included only patients who were ultimately admitted to an ICU.

### Measurements, calculations, and classification of metabolic acidosis

All samples were analyzed in the hospital's central laboratory (Vitros 950; Ortho-Clinical Diagnostics, Raritan, NJ, USA) or blood gas laboratory (Radiometer-ABL 725; Radiometer, Copenhagen, Denmark) for processing. Serum samples were collected in a serum separator tube and sent to the central laboratory for processing. Blood gas samples were collected in a sodium heparin syringe and sent to the blood gas laboratory. Na^+^, K^+^, and Cl^- ^were measured in the central laboratory with the use of a direct ion-selective electrode technique. Albumin was measured with a bromocresol dye colorimetric technique, Mg^2+ ^by a formazan dye derivative colorimetric technique, phosphate by a phosphomolybdate complex colorimetric technique, and Ca^2+ ^by an Arsenazo III dye colorimetric technique. Partial arterial CO_2 _tension and lactate were measured and base excess was calculated on the Radiometer-ABL 725 analyzer in the blood gas laboratory.

### Quantitative acid–base calculations

Quantitative acid–base analysis was performed as described by Stewart [18] and later modified by Figge and colleagues [16] to account for the effects of plasma proteins. The essence of this approach revolves around the concept that there are only three independent variables of acid–base status: partial pressure of carbon dioxide (pCO_2_), strong ion difference (SID), and the total amount of weak acids and proteins in plasma, mainly comprising albumin and phosphates. The SID is the net charge balance of all strong ions present, where a 'strong ion' is one that is completely (or almost completely) dissociated. This is also described as the apparent SID, SID_a_, and can be represented by the following equation (all concentrations in mEq/l):

SID_a _= (Na^+ ^+ K^+ ^+ Ca^2+ ^+ Mg^2+^) - (Cl^- ^+ lactate^-^)

However, this does not account for the role of weak acids in the balance of electrical charges in plasma water. This is can be expressed through the calculation of the effective SID (SID_e_). The formula for SID_e _as determined by Figge and colleagues. [16] is as follows:

SID_e _= 2.46 × 10^-8 ^× pCO_2 _(mmHg)/10^-pH ^+ (albumin (g/l)) × (0.123 × pH - 0.631) + (phosphate (mg/dl)) × (0.309 × pH - 0.469)

This formula accounts quantitatively for the contribution of weak acids to the electrical charge equilibrium in plasma. The difference between SID_a _and SID_e _should range from 0 to 2 mEq/l in healthy patients [17,19], by following the law of electroneutrality. If there are unexplained charges present, they will be represented by a 'gap' between the SID_a _and SID_e_; this is known as the strong ion gap (SIG) [17] and is represented by the following formula:

SIG = SID_a _- SID_e_

The traditional anion gap, (Na^+ ^+ K^+^) - (Cl^- ^+ HCO_3_^-^), does not account for the effect of weak acids, mostly albumin, which is commonly abnormal in critical illness.

We then classified patients into four groups depending on the anion contributing to most acidosis: no metabolic acidosis defined by SBE ≥ -2 mEq/l; lactic acidosis in which lactate accounted for more than 50% of SBE; SIG acidosis in which the SIG (unmeasured ions) accounted for more than 50% of SBE (and not lactic acidosis); and hyperchloremic acidosis defined as SBE < -2 mEq/l not explained by lactate or SIG. An absolute level of chloride was not used for this definition, because it is the relative relationship between the sodium and chloride concentrations that contribute to SID_a _and thus one of the independent variables comprising acid–base equilibria [20]. Thus, if there is a metabolic acidosis present and the SIG or lactate does not make up most of the acid load, the only strong ion left is chloride.

### Statistical analyses

We compared hospital mortality and length of stay for patients in each group; by using logistic regression we identified variables independently associated with mortality in the overall cohort. Unadjusted mortality between groups of metabolic acidosis was analyzed by χ^2 ^test. Length-of-stay analysis was performed by analysis of variance (MedCalc^® ^version 7.0.1.0). A logistic regression model was used to identify independent predictors of mortality. The following variables were explored and selected by stepwise selection in SAS 8.2 PROC LOGISTIC: age, Na^+^, K^+^, Cl^-^, HCO_3_^-^, Mg^2+^, Ca^2+^, phosphate, albumin, lactate, pCO_2_, partial pressure of oxygen (pO_2_), pH, SBE, SIG, SID, and corrected anion gap (AGc) (all treated as continuous variables), and ICU type and gender (treated as categorical variables). Corresponding dummy variables were created for the categorical variables as necessary. The stepwise logistic regression analysis was conducted automatically (variables were entered in the order of relationship to mortality). Finally, we compared SIG and AGc by Bland–Altman analysis.

## Results

**Figure 1:**
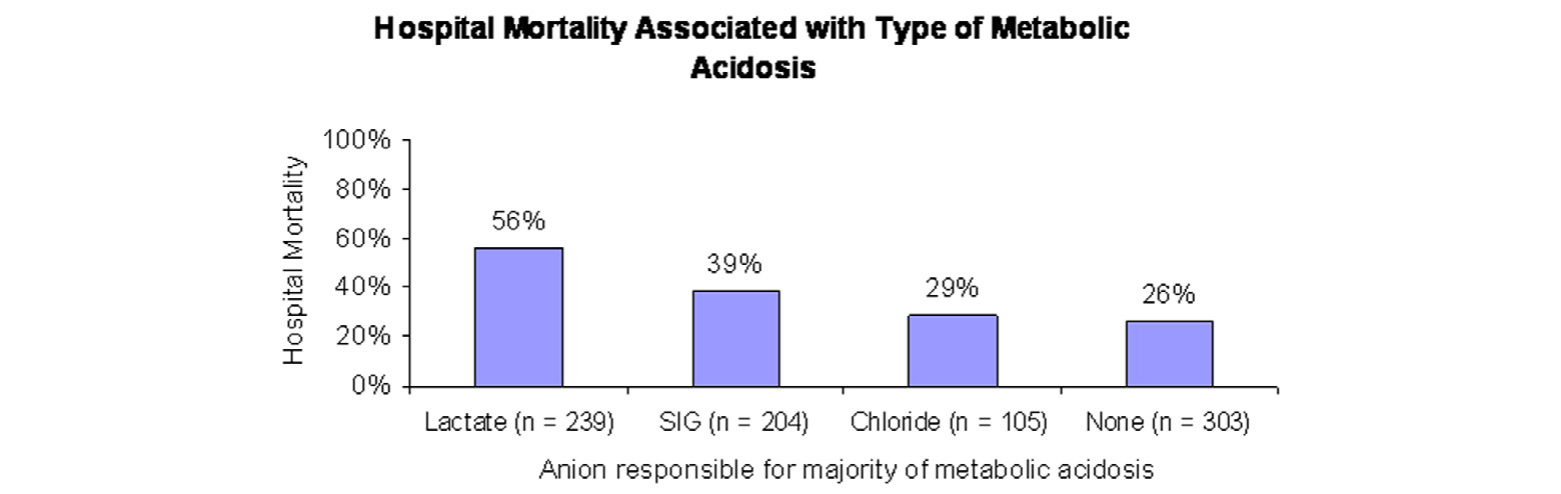
Mortality associated with the major ion contributing to the metabolic acidosis. Hospital mortality associated with the various etiologies of metabolic acidosis (standard base excess (SBE) < -2). Mortality percentage is mortality within each subgroup, not a percentage of overall mortality. 'Lactate' indicates that lactate contributes to at least 50% of the SBE; 'SIG', SIG contributes to at least 50% of SBE (and not lactate); 'hyperchloremic', absence of lactate or SIG acidosis and SBE < -2; 'none', no metabolic acidosis (SBE ≥ -2 mEq/l). SIG, strong ion gap. *p *< 0.001 for the four-group comparison.

We identified a total of 9,799 ICU admissions during the 18 months. This group of patients had an overall mortality of 14.1%, an average hospital length of stay of 12 days and an ICU length of stay of 5.8 days. We then identified 851 patients that fitted entry criteria and, of these, 548 patients (64%) had metabolic acidosis defined by SBE < -2 mEq/l. Among patients with metabolic acidosis, lactic acidosis was the most common (*n *= 239; 44%) followed by SIG acidosis (*n *= 204; 37%). Hyperchloremic acidosis was least common (*n *= 105; 19%). Although length of stay in hospital (32 ± 3 days; mean ± SD) and in ICU (19 ± 4 days) did not vary significantly between these subgroups of metabolic acidosis or between acidotic and non-acidotic patients, they did represent a cohort of patients requiring substantially longer lengths of stay than all ICU admissions. Overall mortality for patients with suspected lactic acidosis was 38%. Mortality was significantly higher for those patients with metabolic acidosis (45%) than for those without (26%, *p *< 0.001, odds ratio 2.29; 95% confidence interval 1.67 to 3.15). Furthermore, mortality varied by major anion associated with the metabolic acidosis (Figure 1).

Demographics and admission diagnostic categories are shown by group in Table 1. All three types of metabolic acidosis seemed to include a similar case mix except that hepatic failure patients were over-represented in the lactic acidosis group and a greater proportion of trauma patients had hyperchloremic acidosis. The results of the univariate analysis and the multivariable logistic regression model are shown in Table 2. This model was developed first by testing for univariate predictors of mortality. Na^+^, K^+^, Cl^-^, HCO_3_^-^, Mg^2+^, Ca^2+^, phosphate, albumin, lactate, pCO_2_, pO_2_, pH, SBE, SIG, SID, AGc, and age were all predictive in this analysis. However, in the final model only lactate, age, SIG, and phosphate levels were independent predictors of mortality. Notably, SBE and pH were not.

**Figure 2:**
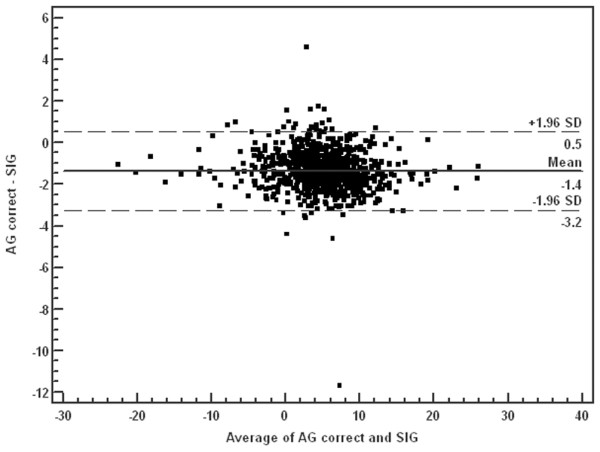
Bland–Altman analysis of corrected anion gap (AGc) and strong ion gap (SIG). Solid line, mean differences; broken lines, ± 1.96 SD. Mean -1.36, SD 0.96, 95% confidence interval (CI) -1.42 to -1.29; lower limit of agreement = -3.24, 95% CI -3.36 to -3.14. Upper limit of agreement = 0.53, 95% CI 0.42 to 0.64. This shows good agreement between AGc and SIG, with only 27 of 851 data points outside 2 SD from the mean.

**Figure 3:**
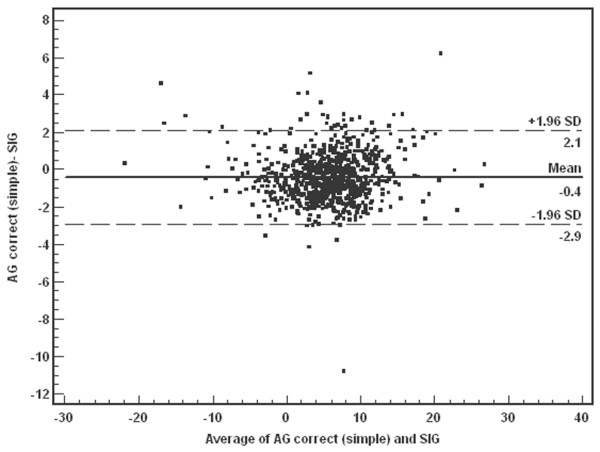
Bland–Altman analysis of simplified corrected anion gap (AGc (simple)) and strong ion gap (SIG). AGc (simple) was calculated in the same was as AGc, but without phosphate. Solid line, mean differences; broken lines, ± 1.96 SD. Mean -0.42, SD 1.28, 95% confidence interval (CI) -0.51 to -0.34. Lower limit of agreement = -2.93, 95% CI -3.08 to -2.79. Upper limit of agreement = 2.08, 95% CI 1.94 to 2.24. This shows good agreement between AGc (simple) and SIG, with only 30 of 851 data points outside 2 SD from the mean.

The SIG calculation (see above) is somewhat cumbersome to use at the bedside [17] and some authors have advocated the use of simpler techniques based on normalizing the anion gap for the serum albumin, phosphate, and lactate concentrations [14,21-23]. Thus, to determine whether AGc could be used in place of SIG, we repeated our multivariable regression using AGc in the model in place of SIG. AGc was calculated as follows: ((Na^+ ^+ K^+^) - (Cl^- ^+ HCO_3_^-^)) - 2.0 × (albumin (g/dl)) - 0.5 × (phosphate (mg/dl)) - lactate (mEq/l) [14]. As expected, SIG and AGc had good agreement by Bland–Altman analysis, with 824 of 851 (96.8%) data points contained within 2 SD of the mean (Figure 2). We also calculated the AGc with an even simpler formula without the use of phosphate, ((Na^+ ^+ K^+^) - (Cl^- ^+ HCO_3_^-^)) - 2.5 × (albumin (g/dl)) - (lactate (mmol/l)) [14], and also found good agreement between SIG and AGc (simple, no phosphate), with 821 of 851 (96.5%) of the data points contained within 2 SD of the mean (Figure 3). Using either formula can be a practical substitute for SIG at the bedside, and the use of AGc did not affect the logistic regression model when substituted for SIG. Even though agreement was good, on closer inspection of our classification system the AGc identified only 172 of the 204 patients (84%) classified as SIG acidosis. This finding may be the result of cut-off values used for the definitions of various metabolic acidosis categories.

## Discussion

Previous studies evaluating the association of metabolic acidosis and outcome in the critically ill have focused on either a specific etiology (for example lactate [24,25]) or a certain degree of acidosis (for example base excess [7,26,27]). Most of these studies had small sample sizes and were, of course, observational in nature. Studies such as these have led to controversy as to whether acidosis is merely a marker for illness severity or whether it is itself in causal pathway of critical illness. Experimental evidence suggests that acidosis itself can influence hemodynamics [11] and innate immunity [13]. Studies also suggest that different acids are associated with different responses [13]. Although no observational study can establish causation, we sought to determine whether different types of metabolic acidosis were associated with different hospital mortality rates. If pH or the degree of metabolic acidosis, as characterized by SBE, are themselves significant determinants of outcome, we would expect to see either a similar mortality across different subtypes of metabolic acidosis or independent associations as determined by multivariable regression. We saw neither. Indeed, the two major findings of our study are that different etiologies of metabolic acidosis are associated with different mortality rates, and that the severity of acidosis, measured by pH or SBE, was not independently associated with hospital mortality after controlling for the causative anion (lactate, chloride, or all others). The finding that pH and SBE were not in the final predictive model may be due to their strong correlation with all forms of metabolic acidoses.

In comparison with chloride, acidosis due to lactate or other anions (SIG) was associated with much higher mortality in hospital (Figure 1). This finding is perhaps not surprising when the causative anion is lactate, because lactic acidosis has been known for some time to be associated with high mortality in this population [24]. However, other anions, measured by SIG, are not clearly associated with poor outcome in ICU patients. Whereas some studies have suggested that SIG is associated with increased mortality in critically ill patients [1,4,28], other studies have not [2,5]. Hucker and colleagues [6] have recently also identified a significant difference in SIG between survivors and non-survivors of patients presenting to the Emergency Department who required hospital admission (4.8 versus 8.2 mEq/l, *p *< 0.001). In our study, the lactate level and SIG were the variables most strongly associated with mortality in critically ill patients suspected of having lactic acidosis. Different ICU populations had similar patterns of mortality associated with the causative anions. Interestingly, measures of severity of acidosis, namely pH and SBE, were not predictive in the final model. This result differs from previous studies [2,5,9,27], perhaps because we sought to quantify all anions (SIG or AGc) for use in our regression model. When we used only chloride and lactate or used the uncorrected anion gap, SBE seemed to be predictive (data not shown), although the model was less predictive overall.

Overall, hyperchloremic acidosis was associated with mortality similar to that of the non-acidotic group (29% versus 26%; *p *= NS). This is perhaps a reassuring finding because many cases of hyperchloremic acidosis are iatrogenic – due to the administration of saline-based intravenous fluids – and can mostly be avoided by resuscitating with a more balanced solution such as lactated Ringer's. However, our methods do not permit us to disentangle the potentially adverse effects of saline-induced acidosis from the potential advantages of fluid administration [29]. Indeed, given that our cohort was that of suspected lactic acidosis, fluid administration might have reversed shock in some patients at the expense of hyperchloremic acidosis. In this way, saline resuscitation might also have converted some patients with hypoperfusion-induced lactic acidosis into hyperchloremia. Against this hypothesis are numerous observations that lactic acidosis in ICU patients usually does not reflect hypoperfusion [30-32]. However, we did not exclude patients whose worst ABG was drawn before ICU admission (though only patients admitted to the ICU were included) and thus our cohort may have included some patients with shock and lactic acidosis and other patients with saline-induced acidosis after resuscitation. Our database does not permit us to test this hypothesis directly and furthermore our sample size, although relatively large, is still insufficient to exclude a clinically significant effect of hyperchloremia on mortality. It is important to keep in mind that chloride is a strong anion and, with regards to plasma, the addition of normal saline increases the value from baseline of chloride more than that of sodium. This change in the ratio of sodium to chloride is what is important [20]. The increase in chloride relative to that of sodium decreases SID, resulting in a decrease in the alkalinity of blood. In this sense, chloride must always be interpreted with the sodium level because they both change with regard to the patient's volume status and the composition of intravenous fluids [33].

Length of stay in hospital and in ICU did not differ significantly between the different groups of metabolic acidosis or even between the groups that did and did not have metabolic acidosis. This may be explained by our selection process (suspicion of lactic acidosis), which most probably selected a cohort that was more unwell than the overall ICU population.

The controversy about conflicting results of six previous outcome studies [1,2,4-6,28] is compounded with our results. Several key limitations of the some of these previous studies were discussed by Rocktaeschel and colleagues. [5] and these authors address several important issues including small sample size [2,5] and low mortality rates [1]. Even though Rocktaeschel and colleagues identified a significant difference in SIG values between survivors and non-survivors, in their logistic regression model there was a strong correlation between SIG, AG, AGc, and standard base excess caused by unmeasured anions. These variables are also strongly linked, making predictive outcomes difficult when all are included in a simple multivariable analysis. Another interesting observation is the role of gelatin-based resuscitation fluid and its effect on SIG. Polygeline-based fluids have been shown to elevate SIG [34], seemingly without increasing mortality. Interestingly, studies that have found that SIG is correlated with outcome were conducted in settings in which gelatin-based fluids were not used [1,4,6,28], whereas studies that found no correlation between SIG and outcomes were conducted in settings in which polygeline-based resuscitation solutions are common [2,5]. Without controlling for resuscitation strategies that increase the amount of unmeasured anions, it is difficult to determine whether SIG is a predictor of mortality.

This study has several limitations. First, as a retrospective study, our database is limited to the variables that were collected for clinical management, and some patients with metabolic acidosis may have been excluded because of missing data. However, these were the data available to clinicians and our study demonstrates that a significant number of patients suspected of having lactic acidosis clinically do in fact have other forms of metabolic acidosis. Second, we could not control for severity of illness between groups. Severity of illness scores (for example Acute Physiology and Chronic Health Evaluation (APACHE)) were recorded only on admission to ICU and only in a subset of patients. Several patients had their highest lactate levels several days into their stay in ICU. However, the use of severity of illness scores are potentially misleading because metabolic acidosis itself might lead to worsening disease severity. Indeed, most severity scoring systems take into account acid–base variables and/or lactate. Thus, for our model we included age as covariate. We also report the admission diagnostic category in Table 2. The nature of our database (laboratory values) also limited our logistic regression model. Ideally we would have included treatment variables (saline and other resuscitation fluid) as well as other clinical variables (hemodynamics). Another limitation involved our initial selection process. The 'suspicion of lactic acidosis' excluded several patients in whom a metabolic acidosis may have been present for other reasons but was not suspected. The categories of metabolic acidosis were used to describe the predominating anion. Rather than using an arbitrary definition of hyperchloremic metabolic acidosis that relies on an absolute value of chloride, we first eliminated all other causes of what has been traditionally classified as an anion gap metabolic acidosis (SIG or lactate). Our classification scheme leaves open the possibility that a combined lactic/SIG acidosis would have been misclassified as hyperchloremic. Conversely, some hyperchloremic cases could have been misclassified as either SIG or lactic acidosis if pre-existing or concomitant metabolic alkalosis was also present, decreasing the apparent impact of chloride. However, these limitations exist with any acid–base classification scheme and given that hyperchloremic acidosis is defined on the basis of 'acidosis without an AG', rather than on the basis of chloride levels, some imprecision is always going to be present.

The association between serum phosphate and mortality was somewhat surprising. Because we did not exclude patients with renal failure, one possible explanation is that elevated phosphate is a marker for renal insufficiency, leading to the positive correlation with mortality. However, if we exclude patients with a phosphate level of more than 5 mg/dl and reanalyze with our regression model, the association between phosphate and mortality remains just as strong. This finding is therefore not explained satisfactorily. Another possible explanation is that increases in phosphate reflect cellular damage. For example, increased serum phosphate in patients with ischemic bowel disease indicates extensive bowel injury [35]. This finding is consistent with the report by Hucker and colleagues. [6] that phosphate (among other biochemical and clinical measurements) was a significant variable in a mortality prediction model of Emergency Department patients who required hospital admission.

The 'SIG' category does not in itself identify an individual causative anion (for example ketones), but rather recognizes the presence of unidentified anions. The term 'SIG' is in itself potentially misleading, because the determinants of SIG include not only strong ions but also weak acids. However, this terminology has been used with increasing frequency and introducing new terminology would most probably confuse rather than clarify. We do not have sufficient information to exclude the effect of acute renal failure on SIG. Comparing the distribution of acidosis subtypes between patients with and without renal dysfunction (defined as a serum creatinine level of more than 2 mg/dl), we found less hyperchloremic and more lactic acidosis in the patients with elevated serum creatinine (Table 3). Interestingly, the proportion of cases defined by elevated SIG was about the same in both groups. These results are similar to those of Rocktaeschel and colleagues. [36], who reported metabolic acidosis and high levels of SIG in patients with acute renal failure. Because patients receiving renal replacement therapy might have normal blood urea nitrogen and creatinine levels at the time of the acidosis, we cannot be sure that they did not have acute renal failure even if the blood urea nitrogen and creatinine were normal. However, even if we knew the patient's native renal function, we could not control for the effect of renal replacement therapy on the SIG. We did not attempt to quantify the presence of ketones or other anions that might be present because these data were usually not available. However, despite these limitations, our study is one of the largest epidemiologic series of critically ill patients with metabolic acidosis.

Many previous studies have demonstrated an association between worsening base excess and mortality. Our study does not refute this. However, our study provides a potential explanation for this finding by identifying the causative anion. Specifically, the base excess does not discriminate between lactate, SIG, and hyperchloremia. If a patient in shock has less than 50% of his base excess explained by lactate and the rest by strong ions other than lactate, then that patient still has an associated 39% mortality according to our data. To our knowledge, these categories have never before been defined and associated with outcomes in this way.

Given the strong and independent association between SIG and mortality in this cohort, it would seem important to monitor SIG clinically. However, the calculation of SIG is cumbersome and although easy enough to derive given a computer or programmable calculator, it seems that correcting the anion gap for albumin, phosphate, and lactate provided a reasonable surrogate for the SIG. Indeed, perhaps even the phosphate correction can be omitted in most cases. However, SIG and AGc show good agreement between the two, and clinically significant differences were rarely observed in our cohort (Figures 2 and 3). In fact, most of the lack of fit is demonstrated at the higher difference values for both the AGc and simplified AGc, meaning that either alternative may produce higher values than the SIG calculation (Figures 2 and 3).

## Conclusion

Not all metabolic acidoses are the same. In our cohort study, each type of metabolic acidosis had a different mortality associated with it. There was increased mortality associated with lactate and unidentified anions (SIG). Metabolic acidosis (both lactic and non-lactic) seems to be associated with high mortality and increased length of stay in hospital and in the ICU. Like that of lactate, monitoring of SIG seems warranted and this can be simplified by the use of an anion gap normalized to the patient's albumin and phosphate concentrations.

## Key messages

• 	Metabolic acidoses resulting from different acids are associated with different outcomes.

• 	Acidosis resulting from lactate and unidentified anions (SIG) were associated with significantly higher hospital mortality than hyperchloremic acidosis.

• 	By multivariate logistic regression, lactate, age, SIG, and phosphate levels were independent predictors of mortality. Notably, SBE and pH were not.

• 	Given the strong and independent association between SIG and mortality in this cohort it seems important to monitor SIG clinically.

• 	AGc (corrected for albumin and phosphate) and SIG show good agreement, and clinically significant differences were rarely observed in our cohort.

## Abbreviations

ABG = arterial blood gas; AGc = corrected anion gap; ICU = intensive care unit; pCO_2 _= partial pressure of carbon dioxide; pO_2 _= partial pressure of oxygen; SBE = standard base excess; SID = strong ion difference; SID_a _= apparent strong ion difference; SID_e _= effective strong ion difference; SIG = strong ion gap.

## Competing interests

The authors declare that they have no competing interests.

## Authors' contributions

KJG was responsible for study concept and design, acquisition of data, analysis and interpretation of data, drafting the manuscript, and statistical analysis. MS performed acquisition of data and provided administrative, technical, or material support. SH conducted analysis and interpretation of data and statistical analysis. JAK was responsible for study concept and design, analysis and interpretation of data, drafting of manuscript, statistical analysis, and administrative, technical, or material support.

## Figures and Tables

**Table 1:** Baseline demographics and admitting diagnostic category by group

Characteristic	Metabolic acidosis: predominant anion			No metabolic acidosis
		
	Lactate	SIG	Chloride	
*n*	239	204	105	303
Age (years)	55.3	60.2	60.8	56.4
Male sex	141 (59)	106 (52)	54 (51)	169 (56)
Admitting diagnostic group				
Hepatic	73 (31)	31 (15)	13 (12)	49 (16)
Infectious	36 (15)	43 (21)	18 (17)	51 (17)
Cardiac	30 (13)	21 (10)	10 (10)	33 (11)
General surgery	18 (8)	10 (5)	9 (9)	43 (14)
Respiratory	14 (6)	25 (12)	8 (8)	26 (9)
Other medical	23 (10)	28 (14)	11 (10)	29 (10)
Trauma	10 (4)	7 (3)	17 (16)	21 (7)
Oncology	11 (5)	16 (8)	8 (8)	18 (6)
Neurological	10 (4)	9 (4)	5 (5)	11 (4)
Vascular surgery	8 (3)	3 (1)	2 (2)	15 (5)
Other surgical	6 (3)	11 (5)	4 (4)	4 (1)
Unknown	0	0	0	3 (1)

SIG, strong ion gap. Numbers in parentheses are percentages.

**Table 2:** Independent predictors of mortality using logistic regression

Variable	Value (mean ± SD)	Odds ratio	95% CI	*p*
Lactate (mEq/l)	5.7 ± 5.8	1.145	1.107–1.184	<0.0001
Age (years)	57.6 ± 16.4	1.023	1.014–1.034	0.0011
SIG (mEq/l)	5.8 ± 5.2	1.065	1.032–1.099	0.0012
Phosphate (mg/dl)	4.0 ± 2.2	1.214	1.118–1.319	<0.0001

With the use of stepwise logistic regression, these variables were identified as independent predictors of mortality (*C *statistic = 0.76). The order of the variables listed reflects their discriminating value in the model. The odds ratio for phosphate seems larger than the other variables because of the narrower distribution of phosphate than that of lactate or SIG. CI, confidence interval; SIG, strong ion gap or unmeasured ions.

**Table 3:** Spectrum of metabolic acidosis in patients with and without renal dysfunction

Acidosis category	Serum creatinine			
	
	<2.0 mg/dl (*n *= 305)		≥2.0 mg/dl (*n *= 211)	
	*n*	%	*n*	%
Chloride (*n *= 105)	76	24.9	22	10.4
SIG (*n *= 204)	105	34.4	83	39.4
Lactate (*n *= 239)	124	40.7	106	50.2

Renal dysfunction was defined as a serum creatinine level of at least 2.0 mg/dl at the time at which the electrolytes were drawn. Acidosis category is described as the predominant anion associated with the metabolic acidosis. SIG, strong ion gap. Percentages are based on column totals. The sum of each row is less than the total *n *because of missing data (no contemporaneous creatinine).
